# Potential use of *Flemingia* (*Flemingia macrophylla*) as a protein source fodder to improve nutrients digestibility, ruminal fermentation efficiency in beef cattle

**DOI:** 10.5713/ajas.20.0214

**Published:** 2020-06-24

**Authors:** Burarat Phesatcha, Bounnaxay Viennasay, Metha Wanapat

**Affiliations:** 1 Department of Agricultural Technology and Environment, Faculty of Sciences and Liberal Arts, Rajamangala University of Technology Isan, Nakhon Ratchasima 30000, Thailand; 2 Tropical Feed Resources Research and Development Center (TROFREC), Department of Animal Science, Faculty of Agriculture, Khon Kaen University, Khon Kaen 40002, Thailand

**Keywords:** Fodder, Nutrients Digestibility, Rumen Fermentation, Feed Resources, Beef Cattle

## Abstract

**Objective:**

This study aimed at studying the potential use of *Flemingia* (*Flemingia macrophylla*) as a protein source fodder to improve nutrients digestibility and ruminal fermentation efficiency in beef cattle.

**Methods:**

Four, Thai native beef cattle were randomly assigned in a 4×4 Latin square design. Four levels of *Flemingia* hay meal (FHM) were used to replace soybean meal (SBM) in the concentrate mixtures in four dietary treatments replacing levels at 0%, 30%, 60%, and 100% of SBM.

**Results:**

The experimental findings revealed that replacements did not effect on intake of rice straw, concentrate and total dry matter (DM) intake (p>0.05). However, the apparent digestibilities of DM, organic matter, crude protein, acid detergent fiber, and neutral detergent fiber were linearly increased up to 100% replacement levels. Moreover, the production of total volatile fatty acids, and propionate concentration were enhanced (p<0.05) whereas the concentration of acetate was reduced in all replacement groups. Consequently, the CH_4_ production was significantly lower when increasing levels of FHM for SBM (p<0.05). Furthermore, rumen bacterial population was additionally increased (p<0.05) while protozoal population was clearly decreased (p<0.05) in all replacement groups up to 100%. In addition, microbial nitrogen supply and efficiency of microbial nitrogen synthesis were enhanced (p<0.05), as affected by FHM replacements.

**Conclusion:**

The findings under this experiment suggest that 100% FHM replacement in concentrate mixture enhanced rumen fermentation efficiency, nutrients digestibilities, bacterial population, microbial protein synthesis, and subsequently reduced CH_4_ production in beef cattle fed on rice straw.

## INTRODUCTION

Commercial concentrate such as protein source is commonly used as a supplement in livestock feeding. However, high cost and uncertain availability has resulted in a search for other feeds. Therefore, farmers have tried to find alternative sources of protein and attempted to use local feed resources to reduce feed costs and improve animal productivity and efficiency [1]. Currently, the legume trees and shrubs have a great potential of high protein leaves as a supplement for ruminants [2]. *Flemingia* (*Flemingia macrophylla*) is native to South and South-East Asia, from India and Sri Lanka, to southern China and Indonesia and is widely distributed in subtropics of Taiwan, Cambodia, Laos, Myanmar, Thailand, Vietnam, Indonesia, Malaysia etc. *Flemingia* has a good tolerance and regrowth after cutting and develops well in soils that are acidic, poorly drained and with low fertility. Legumes, such as *Flemingia*, have a high protein content ranging from 16.9% to 23.7% of dry matter (DM) [3] and have a tannin content ranging from 2.4% to 3.3% [4]. Kang et al [5] also showed that *Flemingia* foliage contained high crude protein (CP) at 25.8% of DM with condensed tannins (CT) at 5.8% of DM. The tropical leguminous shrub leaves contained high level of CP, minerals, as well as phytonutrients such as tannins, and saponins (SP) which are particularly interesting due to their effects in the rumen. Some studies have indicated that high CT levels in the diet would reduce feed intake, reduce protein degradation and improve cell wall digestion in the rumen [6,7]. CT can inhibit the growth or development of methanogens or protozoa in the rumen through bactericidal or bacteriostatic action [8].

Alternatively, *Flemingia* supplementation could decrease rumen protein degradation thus increasing the supply of rumen degradable protein in the lower the gut. Further, the experiment showed that FMH supplementation could improve the fermentation process in *in vitro* and reduce the production of methane [5]. In previous studies, positive effects of CT and SP on growth performance and milk production have been shown. This included a protective influence on rumen dietary protein that enhanced duodenal absorption as well as improving the acetate to propionate ratio in rumen fluid [9]. Moreover, Phesatcha et al [10] reported that FHM supplement in dairy steers affected the nutrient digestibility and improved ruminal fermentation, especially propionate. In this sense, the aim of the research was to determine the potential use of *Flemingia* (*Flemingia macrophylla*) as a protein source fodder to improve nutrients digestibility, ruminal fermentation efficiency in beef cattle.

## MATERIALS AND METHODS

### Experimental animals and management

This study was approved by the Animal Care and Use Committee of Khon Kaen University.

Four, Thai native beef cattle with an initial weight of 200± 10 kg were randomly allocated to a 4×4 Latin square design with 4 dietary treatments. Animals received the diets with various levels of FHM at 0%, 30%, 60%, and 100% of DM, as a replacement for soybean meal (SBM), respectively. All animals were fed rice straw (RS) *ad libitum* and additional concentrate was supplemented at 0.5% of body weight (BW). Table 1 presents data of chemical compositions of concentrate, RS, and nutrient composition. Before starting the treatments, the animals were injected with vitamin AD_3_E.

This research was conducted for four periods of 3 weeks for each period. The first two weeks was an adaptation phase, while the last week sampling phase. The cows were fed with free water and mineral lick blocks twice a day at 0700 h and 1600 h. In the first 14 days, intake was measured and total urine, and fecal collection remained for the last 7 days.

### Samples collection and chemical analyses

All sampling of feed, rumen fluid, and blood samples followed the procedures as described in Wanapat et al [11] including rumen methane estimation. The samples were divided into two parts; the first portion was used for DM analysis while the second part was to evaluate the components of nutrients such as CP, and ash by the AOAC method [12]. Fiber contents such as acid detergent fiber (ADF) and neutral detergent fiber (NDF) were analyzed according to Van Soest et al [13]. CT of *Flemingia* were analyzed by Burns [14] as modified according to report by Poungchompu et al [15].

On the last day of each period, rumen fluid and blood samples were taken at 0 and 4 hours after morning feeding. Rumen fluid was measured for pH immediately, later analyzed for ammonia nitrogen (NH_3_-N) by AOAC method [12] and volatile fatty acids (VFAs) according to Samuel et al [16]. Estimation of ruminal methane production was done using VFA proportions by CH_4_ production = 0.45(acetate, C_2_)− 0.275(propionate, C_3_)+0.4(butyrate, C_4_) [17]. The second part of the filtered fluid sample was for measurement of the rumen microbial population, including bacteria, protozoa, and fungi using the method of Galyean [18]. The second portion was fixed in a sterilized 0.9 percent saline solution with 10 percent formalin solution and then analysed using the methods of total direct counting of bacteria, protozoa, and fungal zoospores [18].

A jugular vein blood sample (about 10 mL) was collected (at the same time as rumen fluid sampling) and analyzed for blood urea nitrogen (BUN) according to the method of Crocker [19]. Fecal and urine samples were collected during the last 7 days of each period using total collection method. The feces were dried at 60°C for 2 or 3 days then were ground and used for nutrient digestibilities (Cyclotech Mill, Tecator, Höganäs, Sweden). The feces were chemically assessed for DM, ash, and CP [12]; NDF and ADF were measured according to the procedure of Van Soest et al [13]. Urine samples were collected and analyzed for allantoin by high performance liquid chromatography [20]. The efficiency of microbial N supply (EMNS) was a good indicator for measurement of the digestible organic matter (OM) to the unit of nitrogen fermented in the rumen [21]. The sum of absorption by microbial purines derivative (PD) was determined from PD excretion based on the relationship derived from the Liang et al [22] equation:

$$\text{Y}=0.12\text{X}+(0.20\;{\text{BW}}^{0.75}).$$

Microbial N supply (MNS) was estimated by Chen and Gomes [21] urinary excretion of PD:

MN (g/d)=70X/(0.116+0.83+1,000)=0.727X,

where X and Y are mmol/d PD absorption and excretion.

The EMNS was determined using the following formula:

EMNS=microbial N (g/d)/DOMR,

where DOMR, digestible OM apparently fermented in the rumen (assuming rumen digestion was 650 g/kg OM of total tract digestion, DOMR = DOMI 0.65, DOMI, digestible OM intake).

### Statistical analysis

All data were subjected to analysis of variance according to a 4×4 Latin square design using the general linear models procedures of SAS version 9.3 Edition 2013 [23]. The results were presented as mean values with the standard error of the means. Treatment trends were statistically compared using orthogonal polynomials. The difference among means with p<0.05 was accepted as statistical differences.

## RESULTS

### Chemical composition of the experimental diets

The data of the concentrate ingredients and chemical composition of the experimental feeds are presented in Table 1. Concentrate mixtures were based on local feed resources containing CP that were similar among treatments. FHM has high concentrations of CP 25.6% and CT 5.6%. RS has a low CP (2.4%) and high content of NDF 75.2% and ADF 46.6%, respectively. In addition, an increasing level of FHM resulted in increased NDF and ADF content in the concentrate.

### Voluntary feed intake, digestibility, and intake of nutrients

Table 2, reports on data of all intakes and the apparent digestibilities (%) of nutrients. Additionally, the apparent digestibilities (DM, OM, CP, NDF, and ADF) were linearly increased from 62.3% to 67.4%, 67.2% to 68.3%, 64.2% to 68.7%, 52.6% to 58.3%, and 45.3% to 52.7% of DM, respectively. The findings suggest that up to 100% SBM supplementation could be replaced by FHM. The low digestibility of FHM was probably due to the CT, which would impact on the rumen degradation of the microorganisms. This phenomenon may be because CT decreases the number of protozoa, and bacteria are the protozoa feed substrate; thus, the population of fibrolytic bacteria will be expected to increase afterwards [24].

### Rumen fermentation characteristics, blood metabolites and microbial populations

As shown in Table 3, the measured rumen variables including the ruminal pH, temperature, NH_3_-N, and BUN were not statistically different among treatments (p>0.05) at all levels when SBM was replaced with FHM, but the VFA profiles were impacted (p<0.05). In addition, the production of total VFA, and propionate proportion were increased, while proportion of acetate reduced in all supplementation groups up to an increase of 100% FHM in the diet. However, CH_4_ estimation was significantly lower with increasing levels of FHM replacing SBM (p<0.05). Furthermore, the bacteria population was the highest values while the protozoal population was decreased in all supplementation groups with FHM replacing SBM at 100% DM (p<0.05). However, the rumen fungal zoospores were not affected by treatments (p> 0.05) when comparing the control group.

### Microbial protein synthesis

Table 4 shows the excretion of urinary PD and EMNS. The results revealed that allantoin excretion and absorption in all treatments were not affected (p>0.05). However, MNS and EMNS were significantly enhanced (p<0.05) when SBM was replaced fully by FMS.

## DISCUSSION

### Chemical composition of the experimental diets

The FHM in the present study contained higher CP and lower CT content than those described by Mui et al [4] and Fagundes et al [25] and could be as a result of the harvesting time and season of harvesting as the dry season would reduce CP and increase CT content. The CT contained in FHM was at an optimal level to form tannin-protein complexes by hydrogen-bonding especially under alkaline pH conditions at pH 3.5 to 7 but will dissociate at pH<3.0 and >8.0 [26].

### Voluntary feed intake, digestibility and intake of nutrients

There were no changes of DM intakes as level of FHM increased in the concentrate mixture, which mean that FHM could completely replace SBM in the concentrate. While, the total DM intakes were in good range for all treatments. Interestingly, the digestibilies of nutrients were significantly enhanced by increasing the level of FHM in replacing SBM in the concentrate. These results could be due to the presence of CT (<6% in the concentrate) which was beneficial in the rumen supporting the microbiome activities, resulting in more C_3_ production. This result agreed with Phesatcha et al [10] reported that FHM supplement in concentrate diets at 150 g/kg, could increase the digestibility of CP and NDF in steers. Fagundes et al [25] reported that results could be influenced by harvesting time and season as harvesting in the dry season will reduce CP and increase CT content of the foliage. Jones and Mangan [26], suggested that CT contained in *Flemingia* was at the optimal level to produce tannin-protein complexes particularly in the presence of alkaline and stable by hydrogen-bonding within the range of pH 3.5 to 7. On the other hand, supplementation of feed containing CT resulted in improved voluntary feed intake, digestion, and metabolism of nutrients that are absorbed [27]. Meanwhile, if CT level were too high (>6% DM), it would reduce feed palatability, digestibility and the productivity of ruminants [28]. Moreover, Min et al [29] demonstrated that intake of CT in the diet of cattle could increase bypass protein, bloat suppression, and daily weight gain in lambs.

### Rumen fermentation characteristics, blood metabolites and microbial populations

The pH values were stable at about 6.6 and the temperature was in normal ranges of about 39°C. As stated by Wanapat and Pimpa [30] that a range of rumen NH_3_-N of 15 to 30 mg/dL was good to support the activities of rumen microorganisms in degrading the roughage in the rumen. While, Beauchemin et al [31] showed that dietary tannins shifted the rumen VFA profile by increasing propionate and narrowed the ratio of acetate to propionate. Furthermore, the rumen population of *Fibrobacter succinogenes* was reduced by high dose of tannins [32]. Moss et al [17] reiterated that rumen fermentation stoichiometric profile would be shifted by the dietary rations and the fermentation end-products could be used to estimate the rumen methane base on the acetate, propionate, and butyrate concentrations. However, Puchala et al [33] stated that feeding CT-containing plants to ruminants resulted in reduced methane emission. Additionally, the data reported by Hess et al [34] revealed the reduction of CH_4_ emission was impacted by supplementation of *Calliandra* and *Flemingia* fodders in an *in vitro* fermentation experiment. In the rumen, CTs may help directly inhibit the growth of methanogens [35]. The hydrogen acceptors from CT can reduce the amount of hydrogen available in the rumen to form CH_4_ [36]. However, Foiklang et al [37] suggested that the use of plant containing CTs had effective action in suppressing methanogens and, hence methane production. This might be due to CT from legume foliage affecting the cell membrane of protozoa. Moreover, Poungchompu et al [15] demonstrated that tannins and saponins reduced protozoa and fungi populations in dairy heifers. Szczechowiak et al [38] reported that *Vaccinium vitis-idaea* containing 4.5% of CT extract could suppress concentration of ruminal methane and resulting in decreased methanogen, bacteria, and protozoal populations. However, Cieslak et al [39] suggested that interaction between tannins and dietary components such as protein and fiber, caused a decrease in the availability of tannins to deal with rumen microbes.

### Microbial protein synthesis

The allantoin excretion and absorption in urine were not affected by the treatments, while MNS and EMNS were significantly enhanced when increasing levels of FHM replaced SBM. Similarly, Phesatcha et al [10] found that MNS was improved when cassava hay was supplemented with FHM in dairy steers. As a result of allantoin absorption, the MNS flow from the rumen ranged from 54.8 to 68.7 g/d, respectively. Waghorn et al [40] recommended that feeding *Lotus corniculatus* improved nitrogen retention and nitrogen absorbed. Furthermore, the rumen microbial protein reaching the lower-gut could be absorbed from 50% to 80% of total absorbable protein in ruminants [41].

## CONCLUSION

The results of the present study show that FHM could be used as a source of protein replacement for SBM which could enhance nutrient digestibility and rumen fermentation efficiency. Furthermore, FHM reduced the ruminal protozoal population and methane production. This study showed that the replacement of SBM by FHM up to 100% in the concentrate mixture was beneficial and improved the nutritive value in ruminant diets.

## Figures and Tables

**Table 1:** Feed ingredients, concentrate mixtures and chemical compositions

Items	Replacement levels of FHM for SBM (% fresh basis)				FHM	RS

	0	30	60	100		
Ingredients (%, fresh basis)						
Cassava chip	60.0	60.0	58.0	58.0		
SBM	20.0	14.0	8.0	0.0		
FLM	0.0	6.0	12.0	20.0		
Rice bran	5.0	4.6	4.0	4.0		
Coconut meal	4.0	4.0	5.0	5.0		
Palm kernel meal	4.0	4.0	5.3	4.8		
Molasses	2.0	2.0	2.0	2.0		
Urea	2.0	2.4	2.7	3.2		
Sulfur	1.0	1.0	1.0	1.0		
Salt	1.0	1.0	1.0	1.0		
Mineral premix	1.0	1.0	1.0	1.0		
Chemical composition						
Dry matter (%)	85.5	84.2	85.3	86.1	84.6	90.2
	---------------------------------------------------------------------% of dry matter---------------------------------------------------------					
Organic matter	92.7	91.4	93.2	94.1	92.3	89.7
Ash	7.3	8.6	6.8	5.9	7.7	10.3
Crude protein	18.3	18.1	18.5	18.8	25.6	2.4
Neutral detergent fiber	18.2	21.3	24.5	26.8	53.1	75.2
Acid detergent fiber	12.3	14.5	16.8	18.9	31.2	46.6
Condensed tannins	-	4.9	5.2	5.3	5.6	-

FHM, *Flemingia* hay meal; SBM, soybean meal; RS, rice straw; FLM, *Flemingia* leaf meal.

**Table 2:** Flemingia as a protein source fodder on voluntary feed intake and nutrient digestibility in beef cattle

Items	Replacement levels of FHM for SBM (% fresh basis)				SEM	Contrasts		
	
	0	30	60	100		Linear	Quadratic	Cubic
Rice straw intake								
kg of DM/d	2.3	2.4	2.4	2.5	0.09	0.07	0.27	0.37
% of BW	1.2	1.3	1.3	1.3	0.05	0.50	0.79	0.87
g/kg BW^0.75^	47.1	49.5	49.7	50.9	1.79	0.43	0.86	0.75
Concentrate intake								
kg of DM/d	0.9	0.8	0.8	0.9	0.16	0.60	0.68	0.47
% of BW	0.5	0.5	0.5	0.5	0.05	0.86	0.83	0.70
g/kg BW^0.75^	18.3	18.2	18.2	18.3	0.02	0.48	0.39	0.27
Total feed intake								
kg of DM/d	3.2	3.2	3.3	3.4	0.09	0.14	0.25	0.16
% of BW	2.0	2.0	2.0	2.1	0.05	0.67	0.93	0.71
g/kg BW^0.75^	71.1	71.1	73.3	75.6	0.17	0.80	0.82	0.67
Apparent digestibility (%)								
Dry matter	62.3^a^	64.1^b^	64.4^b^	67.4^c^	0.49	<0.05	0.31	0.48
Organic matter	67.2^a^	67.6^a^	68.1^b^	68.3^b^	0.76	<0.05	0.45	0.39
Crude protein	64.2^a^	66.7^b^	68.1^c^	68.7^c^	0.60	<0.05	<0.05	0.03
Neutral detergent fiber	52.6^a^	54.8^b^	58.2^c^	58.3^c^	0.35	<0.05	<0.05	0.69
Acid detergent fiber	45.3^a^	46.5^a^	50.1^b^	52.7^c^	0.74	<0.05	<0.05	0.82

FHM, Flemingia hay meal; SBM, Soybean meal; SEM, standard error of the means; DM, dry matter; BW, body weight.

a–c Means in the same row with different superscripts differed (p<0.05).

**Table 3:** Flemingia as a protein source fodder on fermentation characteristics, blood urea nitrogen and microbial population in beef cattle

Items	Replacement levels of FHM for SBM (% fresh basis)				SEM	Contrasts		
	
	0	30	60	100		Linear	Quadratic	Cubic
Ruminal pH	6.5	6.6	6.6	6.6	0.81	0.78	0.85	0.82
Temperature (°C)	39.0	39.1	38.6	38.9	0.40	0.44	0.82	0.46
Total VFAs (mg/dL)	105.0^a^	108.3^ab^	110.1^ab^	112.7^b^	1.32	<0.05	0.35	0.67
Molar of VFAs (%)								
Acetic acid (C_2_)	67.6^b^	65.8^a^	65.3^a^	64.7^a^	0.36	<0.05	0.71	0.88
Propionic acid (C_3_)	22.2^a^	23.1^a^	25.0^b^	25.4^b^	0.16	<0.05	0.35	0.73
Butyric acid (C_4_)	10.2	11.1	9.7	10.0	0.17	0.45	0.62	0.75
CH_4_ estimation (mmol/100 mol)1)	28.4^b^	27.7^b^	26.4^a^	26.1^a^	0.18	<0.05	0.74	0.83
Ammonia-nitrogen (mg %)	17.2	16.2	16.8	17.8	1.32	0.21	0.86	0.45
Blood urea nitrogen (mg/dL)	13.6	13.1	13.3	13.4	1.43	0.63	0.56	0.85
Rumen microbe population (cell/mL)								
Bacteria (×10^11^)	10.2^a^	11.6^b^	13.0^c^	14.3^c^	0.30	<0.05	0.46	0.41
Protozoa (×10^6^)	8.9^c^	6.5^b^	3.7^a^	3.5^a^	0.46	<0.05	0.25	0.67
Fungi (×10^5^)	2.5^a^	2.9^b^	3.1^b^	3.5^b^	0.26	<0.05	0.28	0.75

FHM, Flemingia hay meal; SBM, soybean meal; SEM, standard error of the means; VFAs, volatile fatty acids.

1) CH_4_, methane production = 0.45 (C_2_)–0.275 (C_3_)+0.4 (C_4_) calculated according to Moss et al [17].

a–c Means in the same row with different superscripts differed (p<0.05).

**Table 4:** Flemingia as a protein source fodder on urinary purine derivatives and microbial protein synthesis in beef cattle

Items	Replacement of levels FHM for SBM (% fresh basis)				SEM	Contrasts		
	
	0	30	60	100		Linear	Quadratic	Cubic
Urinary purine derivatives (mmol/d)								
Allantoin excretion	30.1	25.5	27.2	27.7	0.47	0.58	0.71	0.92
Allantoin absorption	75.3	85.2	92.6	94.5	0.52	0.41	0.93	0.76
MNS (g/d)	54.8^a^	62.0^b^	67.5^b^	68.7^b^	1.25	<0.05	0.26	0.56
EMNS (g/kg OMDR)	23.8^a^	28.2^b^	32.1^b^	32.7^b^	0.30	<0.05	0.08	0.35

FHM, Flemingia hay meal; SBM, soybean meal; SEM, standard error of the mean; MNS, microbial nitrogen supply; EMNS, efficiency of microbial nitrogen synthesis; OMDR, digestible organic matter apparently fermented in the rumen.

ab Means in the same row with different superscripts differed (p<0.05).

## References

[1] Guglielmelli A, Calabrò S, Cutrignelli M, Crovetto GM (2010). *In vitro* fermentation and methane production of fava and soy beans. Energy and protein metabolism and nutrition.

[2] van Man N, van Hao N, minh Tri V (1995). Biomass production of some leguminous shrubs and trees in Vietnam. Livest Res Rural Dev.

[3] Andersson MS, Peters M, Schultze-Kraft R, Franco LH, Lascano CE (2006). Phenological, agronomic and forage quality diversity among germplasm accessions of the tropical legume shrub *Cratylia argentea*. J Agric Sci.

[4] Mui NT, Ledin I, Udén P, Van Binh D (2001). Effect of replacing a rice bran–soya bean concentrate with Jackfruit (*Artocarpus heterophyllus*) or *Flemingia* (*Flemingia macrophylla*) foliage on the performance of growing goats. Livest Prod Sci.

[5] Kang S, Wanapat M, Phesatcha K (2017). Using krabok (*Irvingia malayana*) seed oil and *Flemingia macrophylla* leaf meal as a rumen enhancer in an *in vitro* gas production system. Anim Prod Sci.

[6] Barry TN, Duncan SJ (1984). The role of condensed tannins in the nutritional value of *Lotus pedunculatus* for sheep: 1. voluntary intake. Br J Nutr.

[7] Pritchard DA, Stocks DC, O’sullivan BM, Martin PR, Hurwood IS, O’Rourke PK (1988). The effect of polyethylene glycol (PEG) on wool growth and liveweight of sheep consuming a mulga (*Acacia aneura*) diet. Proc Aust Soc Anim Prod.

[8] Liu H, Vaddella V, Zhou D (2011). Effects of chestnut tannins and coconut oil on growth performance, methane emission, ruminal fermentation, and microbial populations in sheep. J Dairy Sci.

[9] Salem AZM, Kholif AE, Elghandour MMY (2014). Influence of oral administration of *Salix babylonica* extract on milk production and composition in dairy cows. Ital J Anim Sci.

[10] Phesatcha B, Wanapat M, Phesatcha K, Ampapon T, Kang S (2016). Supplementation of *Flemingia macrophylla* and cassava foliage as a rumen enhancer on fermentation efficiency and estimated methane production in dairy steers. Trop Anim Health Prod.

[11] Wanapat M, Gunun P, Anantasook N, Kang S (2014). Changes of rumen pH, fermentation and microbial population as influenced by different ratios of roughage (rice straw) to concentrate in dairy steers. J Agric Sci.

[12] AOAC (2012). Official methods of analysis.

[13] Van Soest PJ, Robertson JB, Lewis BA (1991). Methods for dietary fiber, neutral detergent fiber, and nonstarch polysaccharides in relation to animal nutrition. J Dairy Sci.

[14] Burns RE (1971). Method for estimation of tannin in grain sorghum. Agron J.

[15] Poungchompu O, Wanapat M, Wachirapakorn C, Wanapat S, Cherdthong A (2009). Manipulation of ruminal fermentation and methane production by dietary saponins and tannins from mangosteen peel and soapberry fruit. Arch Anim Nutr.

[16] Mathew S, Sagathevan S, Thomas J, Mathen G (1997). An HPLC method for estimation of volatile fatty acids in ruminal fluid. Indian J Anim Sci.

[17] Moss AR, Jouany JP, Newbold J (2000). Methane production by ruminants: its contribution to global warming. Ann Zootech.

[18] Galyean ML (1989). Laboratory procedure in animal nutrition research.

[19] Crocker CL (1967). Rapid determination of urea nitrogen in serum or plasma without deproteinization. Am J Med Technol.

[20] Chen XB, Kyle DJ, Ørskov ER (1993). Measurement of allantoin in urine and plasma by high-performance liquid chromatography with pre-column derivatization. J Chromatogr B Biomed Sci Appl.

[21] Chen XB, Gomes MJ (1992). Estimation of microbial protein supply to sheep and cattle based on urinary excretion of purine derivatives - an overview of the technical details.

[22] Liang JB, Matsumoto M, Young BA (1994). Purine derivative excretion and ruminal microbial yield in Malaysian cattle and swamp buffalo. Anim Feed Sci Technol.

[23] SAS Institute Inc (2013). SAS/STAT 9.3 User’s guide: statistics.

[24] Ampapon T, Phesatcha K, Wanapat M (2019). Effects of phytonutrients on ruminal fermentation, digestibility, and microorganisms in swamp buffaloes. Animals.

[25] Fagundes GM, Modesto EC, Fonseca CEM, Lima HRP, Muir JP (2014). Intake, digestibility and milk yield in goats fed *Flemingia macrophylla* with or without polyethylene glycol. Small Rumin Res.

[26] Jones WT, Mangan JL (1977). Complexes of the condensed tannins of sainfoin (*Onobrychis viciifolia* scop.) with fraction 1 leaf protein and with submaxillary mucoprotein, and their reversal by polyethylene glycol and pH. J Sci Food Agric.

[27] Kamra DN, Agarwal N, Chaudhary LC (2006). Inhibition of ruminal methanogenesis by tropical plants containing secondary compounds. Int Congr Ser.

[28] Naumann HD, Tedeschi LO, Zeller WE, Huntley NF (2017). The role of condensed tannins in ruminant animal production: advances, limitations and future directions. Rev Bras Zootec.

[29] Min BR, Barry TN, Attwood GT, McNabb WC (2003). The effect of condensed tannins on the nutrition and health of ruminants fed fresh temperate forages: a review. Anim Feed Sci Technol.

[30] Wanapat M, Pimpa O (1999). Effect of ruminal NH_3_-N levels on ruminal fermentation, purine derivatives, digestibility and rice straw intake in swamp buffaloes. Asian-Australas J Anim Sci.

[31] McMahon LR, McAllister TA, Berg BP (2000). A review of the effects of forage condensed tannins on ruminal fermentation and bloat in grazing cattle. Can J Plant Sci.

[32] Beauchemin KA, McGinn SM, Martinez TF, McAllister TA (2007). Use of condensed tannin extract from quebracho trees to reduce methane emissions from cattle. J Anim Sci.

[33] Puchala R, Min BR, Goetsch AL, Sahlu T (2005). The effect of a condensed tannin-containing forage on methane emission by goats. J Anim Sci.

[34] Hess HD, Monsalve LM, Lascano CE, Carulla JE, Diaz TE, Kreuzer M (2003). Supplementation of a tropical grass diet with forage legumes and Sapindus saponaria fruits: effects on *in vitro* ruminal nitrogen turnover and methanogenesis. Aust J Agric Res.

[35] Patra AK, Saxena J (2010). A new perspective on the use of plant secondary metabolites to inhibit methanogenesis in the rumen. Phytochemistry.

[36] Naumann HD, Tedeschi LO, Muir JP, Lambert BD, Kothmann MM (2013). Effect of molecular weight of condensed tannins from warm-season perennial legumes on ruminal methane production *in vitro*. Biochem Syst Ecol.

[37] Foiklang S, Wanapat M, Norrapoke T (2016). Effect of grape pomace powder, mangosteen peel powder and monensin on nutrient digestibility, rumen fermentation, nitrogen balance and microbial protein synthesis in dairy steers. Asian-Australas J Anim Sci.

[38] Szczechowiak J, Szumacher-Strabel M, El-Sherbiny M, Pers-Kamczyc E, Pawlak P, Cieslak A (2016). Rumen fermentation, methane concentration and fatty acid proportion in the rumen and milk of dairy cows fed condensed tannin and/or fish-soybean oils blend. Anim Feed Sci Technol.

[39] Cieslak A, Zmora P, Matkowski A (2016). Tannins from *Sanguisorba officinalis* affect *in vitro* rumen methane production and fermentation. J Anim Plant Sci.

[40] Waghorn GC, Ulyatt MJ, John A, Fisher MT (1987). The effect of condensed tannins on the site of digestion of amino acids and other nutrients in sheep fed on *Lotus corniculatus* L. Br J Nutr.

[41] Firkins JL, Yu Z, Morrison M (2007). Ruminal nitrogen metabolism: perspectives for integration of microbiology and nutrition for dairy. J Dairy Sci.

